# Relationship Between Tobacco Retailers’ Point-of-Sale Marketing and the Density of Same-Sex Couples, 97 U.S. Counties, 2012

**DOI:** 10.3390/ijerph120808790

**Published:** 2015-07-28

**Authors:** Joseph G. L. Lee, Adam O. Goldstein, William K. Pan, Kurt M. Ribisl

**Affiliations:** 1 Department of Health Behavior, Gillings School of Global Public Health, The University of North Carolina at Chapel Hill, Chapel Hill, NC 27599, USA; E-Mail: kurt_ribisl@unc.edu; 2 Department of Family Medicine, School of Medicine, The University of North Carolina at Chapel Hill, CB 7595, Chapel Hill, NC 27599, USA; E-Mail: aog@med.unc.edu; 3 Lineberger Comprehensive Cancer Center, The University of North Carolina at Chapel Hill, Chapel Hill, NC 27599, USA; 4 Duke Global Health Institute, Duke University, Box 90519, Durham, NC 27708, USA; E-Mail: william.pan@duke.edu; 5 Nicholas School of Environment, Duke University, Box 90519, Durham, NC 27708, USA

**Keywords:** homosexuality, marketing, smoking, residence characteristics, commerce, health status disparities

## Abstract

The reasons for higher rates of smoking among lesbian, gay, and bisexual (LGB) people than among heterosexual people are not well known. Research on internal migration and neighborhood selection suggests that LGB people are more likely to live in neighborhoods where the tobacco industry has historically targeted their marketing efforts (lower income, more racial/ethnic diversity). We used multi-level models to assess the relationship between the rate of same-sex couples per 1000 coupled households and 2012 marketing characteristics of tobacco retailers (*n* = 2231) in 1696 census tracts in 97 U.S. counties. We found no evidence of tobacco marketing at retailers differing by same-sex couple rates in census tracts with the exception of three findings in the opposite direction of our hypotheses: a small, significant positive relationship for the rate of same-sex male couples and the price of Newport Green (mentholated) cigarettes. For male and female same-sex couples, we also found a small negative relationship between tobacco advertisements and same-sex household rate. Tobacco retailers’ tobacco marketing characteristics do not differ substantially by the rate of same-sex couples in their neighborhood in ways that would promote LGB health disparities. Further work is needed to determine if these patterns are similar for non-partnered LGB people.

## 1. Introduction

Lesbian, gay, and bisexual (LGB) people are at much higher risk of tobacco use than their straight counterparts [1]. In addition to more than 50% higher smoking prevalence than for straight people [2], LGB people are more likely to smoke menthol cigarettes, flavored little cigars, filtered little cigars, and use e-cigarettes than heterosexual people [2,3,4,5]. Data for transgender populations are scarce [6], although similar disparities likely exist [7]. The reasons for these disparities are only partially known, and research has focused primarily on the role of discrimination, stigma, and stress [8]. Researchers have also suggested that the role of LGB bars as safe community spaces may promote tobacco use [9,10], that the media environment may contribute as tobacco use is normative in the LGB print press and in LGBT-themed movies [11,12,13], and that tobacco industry marketing targeted directly at LGB communities contributes to disparities [14,15,16].

In one of the tobacco industry’s plans to market to gay men titled Project Sub-Culture Urban Market (SCUM), RJ Reynolds Tobacco Co. (Winston-Salem, NC, USA) planned to make its products and their marketing ubiquitous in a neighborhood considered to be a gay enclave, San Francisco’s Castro [14,17]. Project SCUM called for better “in store presence”, better “store front presence”, and “consistent POS[point-of-sale]/PDI placements”, with an objective to “[p]enetrate fragmented/nontraditional outlets to increase Camel’s Distribution and presence” [17]. Thus, in one of the clearest examples of tobacco industry targeted marketing to LGB people, Reynolds sought to make its marketing ubiquitous in tobacco retailers in a gay neighborhood.

In studies of the etiology of LGB health disparities in tobacco [8], neighborhood-level marketing to LGB people has been largely ignored although some research has examined political and social environments at the county level and school level in relation to LGB youth smoking [18]. However, there is emerging demographic evidence that internal migration of LGB people within the United States results in the concentration of same-sex couples (We use the terminology “same-sex couples” to describe patterns available from the U.S. Census, which does not report on individual sexual orientation. When discussing the literature or conceptual issues, we use LGB.) in certain types of neighborhoods, in regional cities, and in places where there are already more same-sex couples [19,20,21]. These patterns are more complex than the common view of migration of LGB people to major cities [22], reveal decision-making regarding migration that is informed by sexual orientation identity [23,24], and indicate that LGB people have, as a population, unique spatial patterning [19,20,21,25]. There are differences in patterning between same-sex female and male couples with men concentrating into fewer more urban neighborhoods and women in more suburban areas [20,25,26]. Indeed, demographic research suggests that same-sex couples often live in lower income and more diverse neighborhoods [20], especially male same-sex couples [25]. Tobacco industry marketing is frequently found at greater volume at retailers in poorer and less White neighborhoods [27,28,29,30,31,32]. Neighborhoods with more Black residents have more menthol marketing [29,33,34], lower menthol prices [33], and more little cigar marketing [35]. E-cigarettes are more available in higher-income neighborhoods and neighborhoods with more White residents [36]. Thus, same-sex couples are, as a population, more likely to live in a more racially/ethnically diverse neighborhood with lower income; these neighborhoods are likely to have disproportionate tobacco market due to industry targeting of neighborhoods with more Black residents and lower-income [27].

Tobacco marketing at the POS is part of a broader marketing effort that is causally related to smoking behaviors [37,38]. Both a National Cancer Institute monograph and the Surgeon General report highlight the importance of POS marketing to tobacco prevention and control [37,39]. The tobacco industry spends the majority (85%) of its reported marketing dollars at the POS in the United States [40,41]. Two systematic reviews have synthesized the evidence of the impact of POS marketing on tobacco-related health behaviors, suggesting sufficient evidence for policy intervention [42,43]. Because tobacco use starts during adolescence [37], POS tobacco marketing is relevant to our understanding of LGB tobacco use disparities through its role in stymied quit attempts. Greater volume of tobacco marketing at retailers in neighborhoods with more same-sex couples could delay quit attempts or make them more likely to fail [42,43,44,45,46,47,48].

We aimed to examine the association between census tracts’ rate of same-sex couple households and tobacco retailers’ marketing. Because this is the first study to explore the rate of same-sex couples in neighborhoods in relation to retailers’ POS marketing, our hypotheses are driven by two competing approaches. First, same-sex couples tend to live in more diverse neighborhoods [20], which are the same neighborhoods that are more likely to be targeted by the tobacco industry [27,28,29]. These neighborhoods have smaller, non-chain stores with more marketing [49,50,51]. Second, same-sex couples have been associated with neighborhood gentrification [52,53] and rising home prices [54], which are associated with less POS tobacco marketing [29,30]. We proposed eight hypotheses based on the first approach, given the weight of demographic evidence [20] but recognizing that this study is the first to investigate this relationship.

Given the LGB tobacco disparities that exist (higher smoking prevalence, higher menthol use, and higher use of flavored little cigars) we hypothesized store marketing characteristics that would contribute to those disparities in hypotheses 1–7. We then hypothesized the likelihood of stores’ sale of e-cigarettes based on evidence that same-sex couples live in more diverse neighborhoods [36]. The rate of same-sex couple households in census tracts is:

**H_1–2_**: Positively associated with the presence of (*H*_1_) promotional offers and (*H*_2_) Newport-specific (*i.e.*, menthol) promotional offers.

***H*_3_**: Not associated with the advertised price of Marlboro Red cigarettes at tobacco retailers.

***H*_4_**: Negatively associated with the price of Newport-brand mentholated cigarettes at tobacco retailers.

***H*_5–6_**: Positively associated with numbers of (*H*_5_) total marketing materials and (*H*_6_) total number of exterior marketing materials.

***H*_7–8_**: Positively associated with (*H*_7_) the likelihood of the sale of flavored cigars and (*H*_8_) negatively associated with the likelihood of the sale of e-cigarettes.

## 2. Experimental Section

### 2.1. Selection of Counties

This study is part of a nationally representative study of point-of-sale (POS) tobacco marketing and the methodology is described elsewhere [36]. Briefly, we randomly selected 100 counties with minimal replacement and with probability proportionate to population size using a Chromy [55] technique in SAS 9.2 (SAS Institute, Cary, NC, USA). This resulted in 100 counties (97 unique) where approximately one-quarter of the U.S. population lives [56].

### 2.2. Tobacco Retailer Sampling Frame

For the 97 counties, retailer address and phone data were purchased from two sources in 2012: North American Industry Classification System (NAICS) Association and ReferenceUSA. We requested lists of stores with primary and/or secondary classification as one of the following: supermarkets and other grocery (except convenience) stores; convenience stores; tobacco stores; gasoline stations with convenience stores; warehouse clubs and supercenters; news dealers and newsstands; beer, wine, and liquor stores; pharmacies and drug stores; discount department stores; and other gasoline stations. Codes were selected for store types most likely to sell tobacco.

Data cleaning was conducted using a cleaning protocol that removed stores with no addresses, removed punctuation and spaces, removed suite numbers, replaced PO boxes, and removed non-street address (e.g., airport stores). The cleaning process included eliminating discount department stores other than Walmart, removing separate stores within Walmarts (e.g., Walmart Bakery), retaining only the top 50 pharmacy chains, and removing stores known to not sell tobacco (e.g., state-owned liquor stores, Aldi, Trader Joe’s, Whole Foods). This was conducted separately for NAICS Association and ReferenceUSA lists. Lists were then merged by ZIP code and address and manually de-duplicated.

As part of data cleaning for in-person data collection regarding marketing at the POS, up to 55 randomly selected stores per county from the cleaned sampling frame of tobacco retailers were initially verified by telephone with up to three callbacks using a standardized phone script and computer-assisted dialing. Telephone verification indicated that a majority of retailers in each county (M = 56%, SD = 9%) included in the merged lists could be confirmed by telephone as tobacco retailers. For each selection of a county, up to 24 phone-verified stores were selected for in-person observation.

### 2.3. POS Marketing Audit

Thirteen data collectors participated in a five-hour, in-person training with practice at local stores. Data collectors then visited each store and conducted an audit of tobacco products and marketing materials from June through October 2012 using an iPad (Apple, Cupertino, CA, USA). We assigned 2346 stores to be visited. Of these 2236 were eligible and data were fully collected at 97% of them. This resulted in 2231 store audits, of which 67 only assessed the store exterior due to refusal for interior data collection (*n* = 55) or temporary closure/construction (*n* = 12). Non-response was more likely to be in alcohol (OR = 3.06, 95% CI: 1.78–5.25) or tobacco stores (OR = 4.79, 95% CI: 2.29–9.57) than a typical store and in neighborhoods (tracts) with more Black residents in 10-percentage point increments (OR = 1.12, 95% CI: 1.01–1.24). We assessed reliability of marketing audits by assigning eight auditors to repeat audits at 166 stores; we calculated inter-rater reliability using Krippendorff’s alpha [57]. Audits were often over a week apart; some variability is expected due to changes in the store environment. When stratified by time, audits with a short retest interval had higher reliability than those with longer intervals. Thus, lower reliability may partially reflect expected rotation of store marketing and promotions. Table 1 shows definitions of marketing materials used and reliability.

**Table 1:** Dependent variables by domain of marketing and inter-rater reliability.

Marketing Type	Response Options	Krippendorff’s α
**Price**		
Advertised price, Marlboro Reds	$XX.XX	0.71
Advertised price, Newport Green (mentholated)	$XX.XX	0.86
Price promotions (a multi-pack discount, a special (*i.e.*, discounted) price, or both), interior or exterior	Yes, No	0.42
Price promotion (a multi-pack discount, a special (*i.e.*, discounted) price, or both) for Newport Green (mentholated), interior or exterior	Yes, No	0.45
**Promotion**		
Total marketing materials, *i.e.*, branded signs, branded displays, branded shelving units, and branded functional items (e.g., change mats, clocks, floor mats)	Count	0.63
Total exterior marketing materials, *i.e.*, branded signs and branded functional items (e.g., trash cans, push/pull door signs)	Count	0.70
**Product**		
Flavored cigars (regular or little) sold	Yes, No	0.63
E-cigarettes sold	Yes, No	0.59

### 2.4. Demographic Data

Data on the concentration of same-sex couples come from the 2010 U.S. Census, which included a question on relationship to the owner or renter of the household (“How is this person related to Person 1?”). By aggregating responses of “Husband or wife” and “Unmarried partner” and comparing to the sex of each person, same-sex couples are computed by the Census Bureau as a subcategory of unmarried partner households, where “an adult who is unrelated to the householder, but shares living quarters and has a close personal relationship with the householder” is present [58]. Census 2010 includes same-sex couples as unmarried partners even when they are legally married and live in states with provisions for same-sex marriage or other legal recognition. An important questionnaire design error has been identified in Census 2010 that caused incorrect reporting of sex in door-to-door data collection by census workers, thereby causing estimates of same-sex couples to exceed the total possible number [59,60]. Table 2 shows definitions of independent variables. To correct for this error, we applied an error-rate correction developed by Gates [61].

We calculated a same-sex couple rate used by Walther *et al.* [62] as shown for male same-sex couple households:

(1) $$\left(\frac{\#\;Male\;Same\;Sex\;Couple\;Households}{\#\;of\;Same\;Sex\;Couple\;Households\;+\;\#\;Opposite\;Sex\;Unmarried\;Couple\;Households\;+\;\#\;Married\;Couple\;Households}\right)\times 1000$$

Census tract demographics come from Census 2010 [63], except for median household income, which is from the American Community Survey, 5-Year Estimates, 2008–2012 [64]. For scaling purposes, percentages were divided by 10 (e.g., 12% = 1.2). Rurality was defined by the U.S. Department of Agriculture’s (USDA) 2013 Urban Rural Continuum Codes [65].

**Table 2:** Independent variables.

Variable	Details
**County Level**	
Rurality	U.S. Department of Agriculture Urban Rural Continuum Codes, 1–9 (in increasing rurality)
**Census-Tract Level**	
Same-Sex Couple Households, Female, per 1000 Coupled Households	Number of female householders with female partner divided by total married and unmarried coupled households and multiplied by 1000
Same-Sex Couple Households, Male, per 1000 Coupled Households	Number of male householders with male partner divided by total married and unmarried coupled households and multiplied by 1000
Percentage African-American Population	Percentage of the total population reporting Black or African-American race alone or in combination with another race, in tens
Percentage Hispanic Ethnicity	Percentage of the total population reporting Hispanic or Latino origin, in 10 s
Median Annual Household Income, Adjusted to 2012 USD	Median household income in the past 12 months, in 2012 inflation-adjusted dollars, in ten-thousands
**Store Level**	
Store Type	Supermarkets (*n* = 399) Convenience stores (*n* = 258) Convenience stores with gas (*n* = 929) Tobacco stores (*n* = 93) Alcohol stores (*n* = 224) Drug stores (*n* = 236) Other (*n* = 90, including Warehouse Clubs, Newsstands, Discount Department Stores, “Other” Gas Stations, and Other Store Types)

### 2.5. Analysis

#### 2.5.1. Selection of Area Unit

Following earlier research [30,34,66,67,68], we elected to conduct all analyses at the census tract level. Additionally, the Census originally developed tracts starting in the early 1900s using local committees to define small relatively stable geographic units that approximated local communities [69]. We determined that census tracts represented the best available geographic level to reflect neighborhood processes and provide a large enough population to also analyze small subgroups (*i.e.*, same-sex couples).

#### 2.5.2. Statistical Approach

Because our data on store audits were for stores located in census tracts within counties, they violate the independence assumption of standard regression procedures. We used a multi-level modeling approach to account for the nested nature of our data. We conducted all preliminary data management in SPSS 22 (IBM, Chicago, IL, USA) and used HLM 7.01 (Scientific Software International, Skokie, IL, USA) to test study hypotheses. Because the sample of counties was drawn with replacement and with probability proportionate to size, we used sampling weights that accounted for county selection and non-response. For advertised price, we used linear models; for counts of marketing, we used generalized linear models with a Poisson distribution; and for dichotomous outcomes we used a binary distribution. For linear models we used full maximum likelihood estimation and for non-linear models we used 9-point adaptive quadrature estimation.

We use different strategies for advertised cigarette prices than for other forms of marketing. Because prices are subject to state and county tax variation, we report a three-level model with random intercepts at the tract and county levels for price variables. However, for other forms of marketing, to facilitate convergence and report consistent models, we report a two-level model with random intercepts at the tract level. Tracts have higher intra-class correlations than counties for these variables. Three-level models showed no substantive differences from two-level models but exhibited convergence problems. (Because this secondary data analysis used a dataset sampled at the county level, weights were only available at the store *or* store and county level. Thus, we did not have weights available for the census tract level. The pattern of results did not differ substantively with weights, without weights, or with control for county population. Nor did it differ substantively between two- and three-level models.)

Based on the previous demography literature, we selected *a priori* a modeling strategy, building same-sex-stratified models for each dependent variable. We chose to stratify female and male same-sex couple rates as there is evidence of different spatial patterning and neighborhood selection by sex of same-sex couples [20]. First, we assessed the association of same-sex couple rates with each of the marketing variables. Second, we included other neighborhood demographic characteristics including racial/ethnic composition, median income, and county rurality. Third, we added retailer store type using weighted-effect coding.

We did not adjust our analyses for multiple comparisons, following Rothman [70] and Poole [71]. Because this is exploratory research, we set critical values to α = 0.05 and used two-tailed tests. The UNC Office of Human Research Ethics exempted the parent study from further review (#12-0765).

## 3. Results and Discussion

We first present results from unadjusted models to assess for the hypotheses. We then discuss the role of covariates in Models 2 and 3.

### 3.1. Price

We could not reject the null of hypotheses 1 (greater presence of promotional offers) and 2 (greater presence of Newport-specific promotional offers) (Table 3 and Table 4) among either female or male same-sex couples. There was, as expected, no significant relationship between same-sex couples and Marlboro cigarettes’ advertised prices (*H*_3_, Table 5). For male same-sex couples and Newport (mentholated) cigarette prices, there was a significant positive association: For every additional male same-sex couple per 1000 coupled households, Newport prices increased by a fraction of a cent ($0.002). That is, for 100 additional same-sex male couples per 1000 couples, prices would be expected to increase by $0.20. This was in the opposite direction of our hypothesis (*H*_4_*)*. Thus, hypothesis 3 was supported for no differences in Marlboro cigarettes, and our findings are in the opposite direction of hypothesis 4 regarding a small but significant association with higher Newport prices. We separately ran all different model 1’s from each table using a dichotomous predictor of same-sex couple household rates (1 = greater than or equal to the 90th percentile, 0 = below 90th percentile). All results were in the same direction. Using this dichotomized predictor of the neighborhoods with the top 10% of same-sex couple rates, stratified by sex of same-sex couples, we also did not find any results in directions consistent with explaining existing disparities in LGB tobacco use. Thus, our results associating rates across neighborhoods in the 97 counties are consistent with results examining just the more classic gay and lesbian enclaves.

### 3.2. Number of Advertisements

For both male and female same-sex couples, the likelihoods of an additional advertisement at retailers were negatively associated with each additional same-sex couple. This was in the opposite direction of our hypothesis. The count of exterior ads was not associated with the same-sex couple rate. Thus we could not reject the null of hypotheses 5 (total marketing materials) or 6 (total exterior ads).

### 3.3. Product Availability

Neither flavored cigars nor e-cigarette sales were associated with the same-sex couple rates, thus we could not reject the null of hypotheses 7–8.

### 3.4. Role of Store Type, Neighborhood Characteristics, and County Rurality

After control for tract-level demographics, the three significant associations did not lose their significance, nor did the addition of control for store type cause the associations to lose their significance (Table 3, Table 4 and Table 5). However, in the third model, the likelihood of sale of flavored cigars was significantly associated with the male same-sex couple rate, OR 0.99 (95% CI: 0.99–1.00).

**Table 3:** Two-level models associating female same-sex couple rate with retailer tobacco marketing characteristics.

	Price Promotion, any ^†^ OR (95% CI)	Price Promotion, Newport OR ^†^ (95% CI)	Marketing IRR ^‡^ (95% CI)	Exterior Marketing ERR ^‡^ (95% CI)	Flavored Cigars ^†^ OR (95% CI)	E-Cigarettes ^†^ OR (95% CI)
**Model 1 (Base)**						
L1: Stores	*n* = 2164	*n* = 2159	*n* = 2164	*n* = 2231	*n* = 2162	*n* = 2157
Intercept (95% CI)	**3.63 (2.93–4.50)**	**0.56 (0.46–0.68)**	**20.55 (19.11–22.10)**	0.97 (0.83–1.13)	**5.94 (4.39–8.03)**	**0.52 (0.44–0.61)**
L2: Tracts	*n* = 1655	*n* = 1652	*n* = 1655	*n* = 1696	*n* = 1654	*n* = 1650
Same-Sex Couple Rate	1.00 (0.98–1.02)	1.01 (0.99–1.03)	**0.99 (0.98–1.00)**	1.00 (0.99–1.02)	1.01 (0.98–1.03)	0.99 (0.97–1.01)
**Model 2 (Base + Neighborhood Characteristics)**						
L1: Stores	*n* = 2164	*n* = 2159	*n* = 2164	*n* = 2231	*n* = 2162	*n* = 2157
Intercept (95% CI)	**5.93 (3.01–11.72)**	1.02 (0.58–1.80)	**38.24 (30.61–47.76)**	**2.96 (1.89–4.63)**	**12.06 (6.25–23.26)**	0.92 (0.57–1.47)
L2: Tracts	*n* = 1655	*n* = 1652	*n* = 1655	*n* = 1696	*n* = 1654	*n* = 1650
Same-Sex Couple Rate	0.99 (0.96–1.01)	0.98 (0.96–1.01)	**0.99 (0.98–1.00)**	0.99 (0.97–1.01)	0.99 (0.97–1.01)	0.99 (0.97–1.01)
% Black (10 s)	1.06 (0.97–1.15)	**1.25 (1.16–1.35)**	**0.96 (0.94–0.99)**	1.03 (0.97–1.08)	**1.13 (1.03–1.24)**	**0.88 (0.82–0.94)**
% Hispanic (10 s)	**0.88 (0.83–0.94)**	**0.92 (0.86–0.97)**	**0.91 (0.89–0.93)**	**0.90 (0.86–0.95)**	0.97 (0.91–1.03)	**0.92 (0.87–0.97)**
Median Income (10 ks)	0.99 (0.93–1.05)	0.96 (0.91–1.01)	**0.94 (0.92–0.96)**	**0.85 (0.82–0.89)**	**0.89 (0.84–0.94)**	1.00 (0.96–1.05)
Rurality Code	0.95 (0.85–1.05)	**0.84 (0.76–0.93)**	1.01 (0.97–1.06)	1.02 (0.94–1.11)	0.96 (0.88–1.06)	**0.87 (0.80–0.94)**
**Model 3 (Base + Neighborhood Characteristics + Store Characteristics)**						
L1: Stores	*n* = 2164	*n* = 2159	*n* = 2164	*n* = 2231	*n* = 2162	*n* = 2157
Intercept (95% CI)	**6.06 (3.01–11.91)**	0.87 (0.48–1.56)	**31.52 (26.17–37.96)**	**1.49 (1.01–2.21)**	**16.50 (7.44–36.57)**	0.72 (0.44–1.20)
Supermarkets	*(weighted-effect coding reference group—see note below)*					
Convenience	0.77 (0.55–1.07)	**1.57 (1.14–2.16)**	**1.13 (1.07–1.18)**	**2.44 (2.10–2.83)**	0.95 (0.64–1.41)	0.90 (0.67–1.20)
Convenience with Gas	**2.59 (2.10–3.19)**	**1.64 (1.41–1.91)**	**1.56 (1.53–1.59)**	**2.83 (2.58–3.10)**	**2.51 (1.97–3.21)**	**1.42 (1.25–1.62)**
Tobacco	1.14 (0.61–2.14)	1.55 (0.89–2.72)	**3.17 (3.00–3.34)**	**7.52 (6.34–8.92)**	**7.47 (1.79–31.25)**	**9.60 (5.30–17.38)**
Alcohol	**0.20 (0.14–0.30)**	**0.40 (0.27–0.60)**	**0.54 (0.51–0.58)**	0.84 (0.70–1.01)	**0.15 (0.10–0.24)**	**0.21 (0.13–0.33)**
Drug	**2.17 (1.40–3.35)**	**2.17 (1.55–3.03)**	**0.58 (0.55–0.62)**	**0.02 (0.01–0.04)**	**1.78 (1.10–2.88)**	**3.49 (2.57–4.74)**
Other	**0.19 (0.11–0.33)**	**0.17 (0.08–0.36)**	**0.65 (0.60–0.70)**	**1.90 (1.57–2.30)**	**0.30 (0.17–0.55)**	**1.95 (1.23–3.11)**
L2: Tracts	*n* = 1655	*n* = 1652	*n* = 1655	*n* = 1696	*n* = 1654	*n* = 1650
Same-Sex Couple Rate	1.00 (0.97–1.02)	0.99 (0.96–1.01)	**0.99 (0.98–1.00)**	1.00 (0.98–1.01)	0.99 (0.97–1.02)	1.00 (0.98–1.02)
% Black (10 s)	1.08 (1.00–1.17)	**1.28 (1.18–1.38)**	0.98 (0.96–1.00)	**1.06 (1.02–1.11)**	**1.18 (1.07–1.31)**	**0.89 (0.83–0.95)**
% Hispanic (10 s)	**0.89 (0.84–0.95)**	**0.93 (0.87–0.99)**	**0.93 (0.91–0.94)**	**0.93 (0.90–0.97)**	1.00 (0.93–1.08)	**0.93 (0.88–0.98)**
Median Income (10,000 s)	1.02 (0.96–1.08)	0.97 (0.92–1.03)	**0.96 (0.95–0.98)**	**0.91 (0.88–0.95)**	**0.90 (0.84–0.96)**	1.01 (0.97–1.07)
Rurality Code	0.91 (0.82–1.01)	**0.83 (0.75–0.92)**	**0.98 (0.95–1.02)**	0.96 (0.90–1.03)	0.92 (0.83–1.03)	**0.86 (0.80–0.94)**
ICC	0.15	0.15	0.51	0.74	0.08	0.07

Notes: Significance at the *p* < 0.05 level is indicated by bolded text. Store type is coded with weighted-effect coding and should be interpreted as the odds of the outcome variable against the typical tobacco retailer. OR = odds ratio; CI = confidence interval; ERR = event rate ratio; ICC = intra-class correlation; ^†^ = Hierarchical generalized linear model (binary); ^‡^ = Hierarchical generalized linear model (Poisson). ICC calculated as *ICC = tau_00_ / (tau_00_ + (pi^2^ / 3))* and should be interpreted as the ICC for a hypothetical latent continuous variable underlying the binary variable. Intercepts are reported as exponentiated and represent odds at value of zero. Price promotions model 2 was estimated with 7 adaptive quadrature points after 9 points would not converge. Weights were applied at L1 and modeled with random tract intercepts.

**Table 4:** Two-level models associating male same-sex couple rate with retailer tobacco marketing characteristics.

	Price Promotion, any ^†^ OR (95% CI)	Price Promotion, Newport OR ^†^ (95% CI)	Marketing IRR ^‡^ (95% CI)	Exterior Marketing ERR ^‡^ (95% CI)	Flavored Cigars ^†^ OR (95% CI)	E-Cigarettes ^†^ OR (95% CI)
**Model 1 (Base)**						
L1: Stores	*n* = 2164	*n* = 2164	*n* = 2164	*n* = 2231	*n* = 2162	*n* = 2157
Intercept (95% CI)	**3.58 (2.98–4.31)**	**0.60 (0.52–0.69)**	**19.89 (18.87–20.97)**	1.04 (0.92–1.17)	**6.43 (4.86–8.50)**	**0.52 (0.47–0.57)**
L2: Tracts	*n* = 1655	*n* = 1655	*n* = 1655	*n* = 1696	*n* = 1654	*n* = 1650
Same-Sex Couple Rate	1.00 (0.99–1.00)	1.00 (0.99–1.01)	**1.00 (0.99–1.00)**	0.99 (0.99–1.00)	1.00 (0.99–1.00)	1.00 (1.00–1.01)
**Model 2 (Base + Neighborhood Characteristics)**						
L1: Stores	*n* = 2164	*n* = 2164	*n* = 2164	*n* = 2231	*n* = 2162	*n* = 2157
Intercept (95% CI)	**4.02 (2.59–6.23)**	0.96 (0.56–1.63)	**35.89 (29.10–44.25)**	**6.29 (4.22–9.38)**	**11.86 (6.32–22.23)**	0.86 (0.55–1.34)
L2: Tracts	*n* = 1655	*n* = 1655	*n* = 1655	*n* = 1696	*n* = 1654	*n* = 1650
Same-Sex Couple Rate	1.00 (0.99–1.00)	0.99 (0.99–1.00)	**1.00 (0.99–1.00)**	**0.99 (0.99–1.00)**	0.99 (0.99–1.00)	1.00 (1.00–1.01)
% Black (10 s)	1.05 (0.99–1.12)	**1.24 (1.15–1.34)**	**0.96 (0.93–0.99)**	1.03 (0.97–1.08)	**1.13 (1.03–1.24)**	**0.87 (0.82–0.93)**
% Hispanic (10 s)	**0.90 (0.86–0.95)**	**0.92 (0.86–0.98)**	**0.91 (0.89–0.93)**	**0.90 (0.86–0.95)**	0.97 (0.91–1.03)	**0.92 (0.87–0.97)**
Median Income (10,000 s)	1.00 (0.95–1.04)	0.96 (0.91–1.01)	**0.94 (0.92–0.96)**	**0.85 (0.82–0.89)**	**0.89 (0.84–0.94)**	1.00 (0.96–1.05)
Rurality Code	0.96 (0.89–1.03)	**0.84 (0.76–0.93)**	1.01 (0.97–1.05)	1.01 (0.94–1.10)	0.96 (0.87–1.05)	**0.87 (0.80–0.94)**
**Model 3 (Base + Neighborhood Characteristics + Store Characteristics)**						
L1: Stores	*n* = 2164	*n* = 2164	*n* = 2164	*n* = 2231	*n* = 2162	*n* = 2157
Intercept (95% CI)	**6.09 (3.21–11.57)**	0.84 (0.48–1.46)	**29.93 (25.11–36.67)**	**1.53 (1.05–2.22)**	**17.24 (8.02–37.03)**	0.71 (0.44–1.15)
Supermarkets	*(weighted-effect coding reference group—see note below)*					
Convenience	0.77 (0.55–1.07)	**1.56 (1.14–2.15)**	**1.12 (1.07–1.18)**	**2.44 (2.10–2.83)**	0.95 (0.64–1.41)	0.90 (0.67–1.20)
Convenience with Gas	**2.58 (2.09–3.18)**	**1.63 (1.40–1.90)**	**1.56 (1.63–1.59)**	**7.51 (6.33–8.91)**	**2.47 (1.94–3.16)**	**1.42 (1.25–1.62)**
Tobacco	1.14 (0.61–2.14)	1.55 (0.89–2.71)	**3.17 (3.00–3.34)**	**2.82 (2.57–3.10)**	**7.43 (1.79–30.84)**	**9.59 (5.30–17.35)**
Alcohol	**0.20 (0.14–0.30)**	**0.40 (0.27–0.60)**	**0.54 (0.51–0.58)**	0.84 (0.70–1.00)	**0.15 (0.10–0.24)**	**0.21 (0.13–0.33)**
Drug	**2.19 (1.42–3.40)**	**2.24 (1.60–3.14)**	**0.59 (0.56–0.62)**	**0.02 (0.01–0.04)**	**1.88 (1.15–3.06)**	**3.52 (2.59–4.80)**
Other	**0.19 (0.11–0.33)**	**0.17 (0.08–0.37)**	**0.65 (0.60–0.70)**	**1.90 (1.57–2.30)**	**0.31 (0.17–0.56)**	**1.96 (1.23–3.13)**
L2: Tracts	*n* = 1655	*n* = 1655	*n* = 1655	*n* = 1696	*n* = 1654	*n* = 1650
Same-Sex Couple Rate	1.00 (0.99–1.00)	0.99 (0.99–1.00)	**1.00 (1.00–1.00)**	1.00 (0.99–1.00)	**0.99 (0.99–1.00)**	1.00 (0.99–1.00)
% Black (10 s)	1.08 (1.00–1.17)	**1.28 (1.18–1.38)**	**0.98 (0.95–1.00)**	**1.06 (1.02–1.11)**	**1.19 (1.07–1.31)**	**0.89 (0.83–0.95)**
% Hispanic (10 s)	**0.89 (0.84–0.95)**	**0.93 (0.87–0.99)**	**0.93 (0.91–0.94)**	**0.93 (0.90–0.97)**	1.00 (0.93–1.07)	**0.93 (0.88–0.98)**
Median Income (10,000 s)	1.02 (0.96–1.08)	0.97 (0.92–1.03)	**0.97 (0.95–0.98)**	**0.91 (0.88–0.95)**	**0.90 (0.84–0.96)**	1.01 (0.97–1.07)
Rurality Code	0.91 (0.82–1.00)	**0.82 (0.75–0.91)**	0.98 (0.95–1.02)	0.96 (0.90–1.02)	0.91 (0.82–1.02)	**0.86 (0.79–0.94)**
ICC	0.15	0.15	0.51	0.74	0.08	0.07

Notes: Significance at the *p* < 0.05 level is indicated by bolded text. Store type is coded with weighted-effect coding and should be interpreted as the odds of the outcome variable against the typical tobacco retailer. OR = odds ratio; CI = confidence interval; ERR = event rate ratio; ICC = intra-class correlation; ^†^ = Hierarchical generalized linear model (binary); ^‡^ = Hierarchical generalized linear model (Poisson). ICC calculated as *ICC = tau_00_ / (tau_00_ + (pi^2^ / 3))* and should be interpreted as the ICC for a hypothetical latent continuous variable underlying the binary variable. Intercepts are reported as exponentiated and represent odds at value of zero. Weights were applied at L1 and modeled with random tract intercepts.

**Table 5:** Three-level models associating same-sex couple rate with retailer tobacco marketing characteristics (97 counties, USA).

	Female		Male	
	Advertised Price, Marlboro $ (SE)	Advertised Price, Newport $ (SE)	Advertised Price, Marlboro $ (SE)	Advertised Price, Newport $ (SE)
**Model 1 (Base)**				
L1: Stores	*n* = 2040	*n* = 1852	*n* = 2040	*n* = 1852
Intercept	**$6.33 (0.17)**	**$6.44 (0.14)**	**$6.33 (0.17)**	**$6.44 (0.14)**
L2: Tracts	*n* = 1610	*n* = 1547	*n* = 1610	*n* = 1547
Same-Sex Couple Rate	<$0.01 (<0.01)	<$0.01 (<0.01)	<$0.01 (<0.01)	**<$0.01 (<0.01)**
L3: Counties	*n* = 97	*n* = 97	*n* = 97	*n* = 97
**Model 2 (Base + Neighborhood Characteristics)**				
L1: Stores	*n* = 2040	*n* = 1852	*n* = 2010	*n* = 1852
Intercept	**$6.75 (0.30)**	**$6.60 (0.28)**	**$6.72 (0.28)**	**$6.60 (0.25)**
L2: Tracts	*n* = 1610	*n* = 1547	*n* = 1610	*n* = 1547
Same-Sex Couple Rate	<$0.01 (<0.01)	**$0.01 (<0.01)**	<$0.01 (<0.01)	**<$0.01 (<0.01)**
% Black (10 s)	$−0.01 (0.01)	**$−0.04 (0.01)**	<$0.01 (0.01)	**$−0.03 (0.01)**
% Hispanic (10 s)	$−0.01 (0.02)	<$0.01 (0.02)	$−0.01 (0.02)	$0.01 (0.02)
Median Income (10,000 s)	$−0.01 (0.01)	$0.02 (0.01)	$−0.01 (0.01)	$0.02 (0.01)
L3: Counties	*n* = 97	*n* = 97	*n* = 97	*n* = 97
Rurality Code	$−0.15 (0.08)	$−0.11 (0.07)	**$−0.16 (0.07)**	$−0.11 (0.07)
**Model 3 (Base + Neighborhood Characteristics + Store Characteristics)**				
L1: Stores	*n* = 2040	*n* = 1852	*n* = 2040	*n* = 1852
Intercept	**$6.75 (0.30)**	**$6.63 (0.27)**	**$6.70 (0.27)**	**$6.61 (0.24)**
Supermarkets	*(weighted-effect coding reference group—see note below)*			
Convenience	<$0.01 (0.03)	**$−0.12 (0.04)**	<$0.01 (0.03)	**$−0.12 (0.03)**
Convenience with Gas	**$−0.04 (0.02)**	**$−0.09 (0.02)**	**$−0.04 (0.02)**	**$−0.09 (0.02)**
Tobacco	**$−0.25 (0.11)**	**$−0.25 (0.08)**	**$−0.25 (0.11)**	**$−0.25 (0.08)**
Alcohol	**$0.22 (0.05)**	**$0.19 (0.05)**	**$0.21 (0.05)**	**$0.19 (0.05)**
Drug	**$−0.35 (0.04)**	**$−0.21 (0.04)**	**$−0.37 (0.04)**	**$−0.23 (0.05)**
Other	**$0.32 (0.07)**	**$0.43 (0.08)**	**$0.33 (0.07)**	**$0.44 (0.08)**
L2: Tracts	*n* = 1610	*n* = 1547	*n* = 1610	*n* = 1610
Same-Sex Couple Rate	<$0.01 (<0.01)	**$0.01 (<0.01)**	**<$0.01 (<0.01)**	**<$0.01 (<0.01)**
% Black (10 s )	$−0.01 (0.01)	**$−0.04 (0.01)**	<$0.01 (0.01)	**$−0.03 (0.01)**
% Hispanic (10 s)	$−0.02 (0.02)	<$0.01 (0.01)	$−0.01 (0.01)	<$0.01 (0.01)
Median Income (10,000 s)	$−0.01 (0.01)	$0.02 (0.01)	<$0.01 (0.01)	$0.02 (0.01)
L3: Counties	*n* = 97	*n* = 97	*n* = 97	*n* = 97
Rurality Code	$−0.15 (0.08)	$−0.11 (0.08)	**$−0.16 (0.07)**	$0.10 (0.07)

Notes: Significance at the *p* < 0.05 level is indicated by bolded text and is reported with robust standard errors. SE = standard error. Intercept is calculated with explanatory variables set at zero. Weight applied at L1 and modeled with random tract and county intercepts. Store type is coded with weighted-effect coding and should be interpreted as the odds of the outcome variable against the typical tobacco retailer.4. Conclusions

## 4. Conclusions

Overall, we found few significant relationships between the rate of same-sex couples in census tracts and eight measures of tobacco marketing in tobacco retailers within those tracts. Nonetheless, our study did find small but significant relationships in unexpected directions, including higher Newport prices for same-sex male couples and fewer ads for both male and female same-sex couples. Although these finding should be replicated in other data sources, it provides no evidence that the origin of LGB tobacco disparities lies in store-level differences in POS tobacco marketing. Nonetheless, our analysis does not take into account differences in retailer density and thus cannot determine if the total volume of marketing *in neighborhoods* is associated with same-sex couple rates. In previous work, we found a small but significant positive association between same-sex couple rates and tobacco retailer density, with markedly higher density among the neighborhoods with the highest rates of same-sex couples [72]. Greater density could indicate greater neighborhood-level tobacco marketing even in the absence of store-level differences in tobacco marketing.

Our results imply that there is no significant difference between marketing in neighborhoods with more same-sex couples compared to neighborhoods with fewer couples. However, in our data, as expected, same-sex couple rates at the tract level were positively associated with the proportion of tract residents reporting Black race (*r_s(n_*_= 1696*)*_ = 0.29, *p* < 0.01; *r_s(n_*_= 1696*)*_ = 0.32, *p* < 0.01) or Hispanic/Latino ethnicity (*r_s(n_*_= 1696*)*_ = 0.11, *p* < 0.01; *r_s(n_*_= 1696*)*_ = 0.17, *p* < 0.01) and negatively associated with median household income (*r_s(n_*_= 1696*)*_ = −0.18, *p* < 0.01; *r_s(n_*_= 1696*)*_ = −0.15, *p* < 0.01), respectively, for female and male same-sex couples. It may be that neighborhoods with higher same-sex couple rates are qualitatively different from those otherwise being targeted by the tobacco industry for their racial/ethnic diversity and lower income. Or, alternatively, processes of neighborhood change including gentrification may have attenuated the relationships between neighborhood demographics and tobacco industry targeting that have previously been documented [27] by changing the composition of neighborhood stores and advertising. It is also possible that growing acceptance of same-sex couples has led to increasing integration of same-sex couples into a broader array of neighborhoods than in previous years. There is some evidence to support this; segregation indices for same-sex couples have declined between the 2000 and 2010 census [21].

As for specific products and types of marketing, flavored cigars are disproportionately used by LGB people [4] as are little filtered cigars [5], and we expected to see a neighborhood-level association with the presence of flavored cigars (regular or little) in tobacco retailers. Not finding this association, we suggest further investigation using other more comprehensive measures of little cigar sales and examination of the potential for flavored little cigars use to be influenced by LGB identity-related preferences to enhance our understanding of neighborhood-level influence of the retail marketing environment.

Fallin *et al.* report that LGB smokers were more likely to use menthol than their heterosexual peers in the National Adult Tobacco Survey [3]. The reasons for this discrepancy are unclear. We note that document research about LGB targeting by the tobacco industry has not reported specific targeting of mentholated products [14,15,73]. Menthol is also disproportionately used by Black and African American smokers [74]; researchers have consistently suggested that this is due to heavy targeting by the tobacco industry [31,74] and linked declines in menthol in Australia to declining marketing [75]. Because we did not find differences in retail marketing of menthol products, other avenues to explain this disparity should be explored. Physiological differences in sensory experience of menthol, which have been suggested by some researchers [76], seem unlikely to explain differences for LGB people. However, menthol use is associated with being health conscious and a desire to quit [77], and we believe future research should examine the role of these as potential reasons for LGB menthol disparities, given gendered and cultural differences in menthol preferences [77].

Last, our research focuses on the neighborhood level of the social-ecological framework and the potential role of POS tobacco marketing therein on LGB tobacco disparities. Further research is needed on other sources of influence on these disparities, such as policies increasing the per-unit cost of tobacco products, other forms of tobacco marketing (e.g., print media, corporate sponsorship), media effects, and differential effects of tobacco use cessation interventions.

### 4.1. Limitations

There are important limitations to this study. First, the census may underestimate same-sex households and only captures information on same-sex couples. Individual LGB people are more likely to live in more urban areas than same-sex couples [26]. Individual sexual orientation data, which is not available in the census, would have strengthened our study, which is not generalizable to LGB individuals.

Second, reliability on some audit measures was low to moderate; we believe this is due to an up to six-week gap between audits. This may reflect expected changes in product promotions (and reliability was negatively related to the length of time between audits); audit questions such as ours generally show good reliability [78]. But lower reliability makes it harder to detect a true effect. Given our largely null findings, this is a cause for concern.

Third, because of the national scope of this study, it was not financially viable to visit retailers who were not known to be tobacco retailers. Our phone verification protocol may have biased our study against the inclusion of smaller, independent retailers who may be in higher minority and lower income neighborhoods [49,50]. Indeed, at the county level, there are differences in phone verification rates by the proportion of county population reporting African American race (*r_s(n_*_= 9*)*_ = −0.21, *p* = 0.04), Hispanic ethnicity (*r_s(n_*_= 97*)*_ = −0.37, *p* < 0.001), and same-sex couple households (*r_s(n_*_= 97*)*_ = −0.28, *p* = 0.01). Phone verification rates do not differ significantly by median county household income (*r_s(n_*_= 97*)*_ = −0.14, *p* = 0.19).

Fourth, this study, because of its sampling strategy, was conducted in largely urban counties. Geographers have noted that assessing differences within higher density LGB areas reduces our ability to see differences across the country [79]. That is, by focusing in urban areas, where there are overall higher concentrations to same-sex couples [20], we may have attenuated our ability to detect differences.

Fifth, we had two limitations from our measures of product marketing. Mentholated products were limited to advertised price of a leading mentholated cigarette brand and the presence of price promotions for that same brand. Future research should examine same-sex couple rates in relation to the volume of marketing for mentholated products. Our measure of flavored cigars was not specific to little cigars and included regular cigars.

Nonetheless, this is one of the largest national audit studies to date, and past systematic reviews [78] have identified no other studies assessing retail tobacco marketing in relation to LGB people or same-sex couples.

### 4.2. Conclusions

In a 2013 systematic review, Blosnich and colleagues noted that the minority stress model is the most frequently used conceptual approach to explaining the origin of LGB tobacco disparities [8]. We attempted to extend this line of research to include spatial patterning of same-sex couples; however, our findings suggest that tobacco industry marketing at the store level is not disproportionately greater in neighborhoods with more same-sex couples. This is not to say existing marketing is not meaningful; even without disproportionate exposure, tobacco industry marketing may have a greater impact on LGB people than heterosexual people possibly due to LGB community appreciation for being recognized [16,80,81]. Further research is needed to assess density in relation to marketing because greater density [72] could cause total ads per neighborhood to be higher even with no store-level differences. Although we hope others will replicate and extend this study, it suggests that the store-level physical marketing environment may play a limited or role or no role in the origin of LGB tobacco disparities, use of mentholated products, use of flavored little cigars, and use of e-cigarettes.
